# Oregon Not-for-Profit Hospital Community Benefit Policy: Trends in Community Benefit Spending

**DOI:** 10.3390/healthcare13131497

**Published:** 2025-06-23

**Authors:** Tatiane Santos, Gary J. Young, Shoou-Yih Lee, Kelsey Owsley

**Affiliations:** 1 Celia Scott Weatherhead School of Public Health and Tropical Medicine, Tulane University, New Orleans, LA 70112, USA; 2 D’Amore-McKim School of Business, Northeastern University, Boston, MA 02115, USA; 3 Bouve College of Health Sciences, Northeastern University, Boston, MA 02115, USA; 4 Northeastern University Center for Health Policy and Healthcare Research, Boston, MA 02115, USA; 5 College of Health Professions, Virginia Commonwealth University, Richmond, VA 23298, USA; 6 Fay W. Boozman College of Public Health, University of Arkansas for Medical Sciences, Little Rock, AR 72205, USA; kmowsley@uams.edu

**Keywords:** community benefit, spending floor, not-for-profit hospitals, social determinants of health, charity care

## Abstract

**Background/Objectives**: Community benefit (CB) obligations by not-for-profit (NFP) hospitals have attracted renewed scrutiny at federal and state levels due to wide variation in CB spending. In 2020, Oregon implemented a CB policy for all NFP hospitals that included requirements to expand patient financial assistance and a hospital-specific minimum CB spending floor. We examined trends in CB spending after the implementation of Oregon’s CB policy. **Methods**: Interrupted time-series analyses to compare hospital CB spending before and after policy implementation. **Results**: Overall, Oregon’s CB policy was not associated with changes in CB spending, except for a 0.2% decrease in the Social Determinants of Health spending (−0.0018; *p* < 0.05). Among hospitals in the first tercile of pre-policy CB spending, Oregon’s policy was associated with a 0.4% decrease in charity care (−0.0041; *p* < 0.05) and a 0.6% increase in subsidized health services spending (0.0063; *p* < 0.05). Hospitals in the second tercile of pre-policy CB spending experienced a 0.7% decrease in subsidized health services (−0.0074; *p* < 0.05). Among frontier hospitals, total CB spending and Medicaid shortfalls increased by 2.9% (0.0292; *p* < 0.10) and 2.2% (0.0220; *p* < 0.10) respectively, while non-frontier hospitals experienced a 0.7% decrease in Medicaid shortfall (−0.0068; *p* < 0.05). Critical access hospitals experienced a 1.3% increase in subsidized health services spending (0.0131; *p* < 0.05). **Conclusions**: Although total CB spending did not change in the two years following Oregon’s CB policy implementation, findings suggest that hospitals may be shifting the composition of their CB spending. Oregon’s CB policy encourages proactive CB spending tailored to community needs, but opportunities exist to fine-tune the policy to boost hospital CB spending. Specifically, planned spending in categories such as charity care may alleviate the increasing burden of medical debt and its financial implications for patients.

## 1. Introduction

The provision of community benefits (CBs) by not-for-profit (NFP) hospitals has been a long-standing policy issue and has attracted renewed scrutiny at the federal level [1,2,3]. Increasingly, legislators are questioning whether or not NFP hospitals make sufficient CB investments to justify their tax exemptions [2,3]. In 2021, the estimated value of this tax exemption was USD 37.4 billion, which represents the amount of money that NFP hospitals should reinvest in their communities [4]. In exchange for these substantial tax savings, NFP hospitals are expected to provide services that improve the well-being of their communities [5].

Several provisions from the Patient Protection and Affordable Care Act (ACA), including Section 9007 that requires hospitals to conduct triennial community health needs assessments, were expected to increase CB spending. However, on a national level, CB spending has remained mostly flat [6]. Meanwhile, many states have adopted regulatory initiatives to exact greater accountability for community benefits from NFP hospitals, with mixed results [7,8,9,10]. While some studies have found modest increases in CB spending and charity care, others have shown little to no change. Oregon House Bill 3076 is a novel approach to regulate hospital community benefit activities, offering a valuable opportunity to assess the effects of an innovative state-level intervention.

In 2020, Oregon House Bill 3076 (i.e., Oregon CB policy) required all NFP hospitals to expand hospital financial assistance and medical debt protections for patients and imposed a hospital-specific minimum CB spending floor [11]. The expansion of hospital financial assistance and medical debt protections were implemented in 2020, while the minimum CB spending floors were implemented in 2021 [11]. The Oregon CB policy is anchored on a rigorous NFP hospital reporting program that adds a layer of accountability and transparency to the new CB requirements [12,13]. The overarching goal is to increase NFP hospital spending across all categories of CB while providing medical debt relief to Oregonians.

Oregon’s approach to setting the minimum CB spending floor stands apart from the other five states (i.e., Illinois, Nevada, Pennsylvania, Texas, and Utah) that have implemented CB spending floors. First, most such states require NFP hospitals to provide charity care or other financial assistance based on a flat percentage of net patient revenues and operating expenses, which may be too blunt an instrument to lead to increased charity care spending [14]. For instance, Texas’ requirement for NFP hospitals to spend at least four percent of net patient revenue in charity care did not, according to at least one study, result in increased charity care spending [15]. Furthermore, the Texas requirement appeared to have had the unintended consequence of decreased charity care spending by NFP hospitals whose charity care spending was above the threshold before the requirement, a phenomenon dubbed “race to the bottom” [15]. Second, some states use ambiguous regulatory language for minimum CB spending floors that may lead to wide variation in interpretation and attenuation of policy impact [14]. For example, Utah requires NFP hospitals to contribute yearly “gifts to the community” in an amount that exceeds the value of its annual property tax liability [14]. Third, some states allow hospitals to choose the approach used to calculate minimum CB spending thresholds which may lead to confusion and partial compliance, as opposed to Oregon’s single approach for all hospitals [14].

The Oregon Health Authority (OHA), which oversees the implementation of the minimum CB spending floor, took proactive steps to gain NFP hospital buy-in related to House Bill 3076 and to address the potential shortcomings from other states’ attempts to set a minimum CB spending floor. The OHA engaged in a participatory process to develop the minimum CB spending floor methodology in consultation with hospitals, health economists, and the public. The hospital-specific spending floors were formulated to be responsive to financial trends and transparent to hospitals and other stakeholders [16]. As such, the Oregon initiative sets an expectation that hospitals enjoying robust financial performance will spend more in CB, with the opposite being true for financially vulnerable hospitals. Hospitals have 30 days to challenge the spending floors based on their own projections or if the spending floors would put them in significant financial hardship [12].

The goal of the Oregon CB policy is to increase NFP hospital spending across all categories of CB, including charity care, shortfalls from Medicaid and other government programs, subsidized health services, community health improvement, cash and in-kind contributions, community building, research, and health profession education. As the policy was novel, no study, to our knowledge, has comprehensively evaluated the impact of the Oregon policy. Prior work has examined the impact of the initial phase of the policy and found it was associated with increased charity care and bad debt spending [17]. In this study, we extend this work by conducting an interrupted time-series analysis to examine Oregon’s overall trends in NFP hospital CB spending and the association between the new CB policy and NFP hospital CB spending using data from 2011 to 2022. Understanding the impact of Oregon’s policy has broad relevance, as many communities continue to debate how best to ensure hospitals contribute meaningfully to community health. Findings from this study can inform policy development in a range of settings and policy areas, including accountable care organizations and medical debt protections.

## 2. Materials and Methods

We used audited hospital financial and CB spending data covering 2011 to 2022 from the OHA’s Hospital Reporting Program. Oregon’s hospitals are required to report yearly financial data and ten categories of CB spending to the OHA. Of the 60 NFP hospitals in Oregon, four were excluded from our analysis because of missing data. We merged county-level data from the Agency for Healthcare Research and Quality’s SDOH database.

The primary study outcomes include hospital spending on total CB, direct patient care (i.e., sum of charity care, shortfalls from Medicaid and other government programs, and subsidized health services), SDOH (i.e., sum of community health improvement, cash and in-kind contributions, community building, and CB operations), research and education (i.e., sum of research and health profession education) and disaggregated spending on charity care, shortfalls from Medicaid and other government programs, and subsidized health services. To account for hospital size, we divided each outcome by a hospital’s total operating expenses (i.e., standardized by hospital financial size). We converted all raw dollar values to 2021 dollars using the consumer price index from the U.S. Bureau of Labor Statistics.

We estimated a series of linear interrupted time-series (ITS) models to evaluate the association between the Oregon CB policy and changes in CB spending. The ITS models allow us to assess trends in CB spending prior to Oregon’s CB policy and then examine changes in those trends that coincide with the first phase of implementation of the CB policy (i.e., 2020). ITS has been widely used in prior research to evaluate the effects of policy changes, public health interventions, and major events such as the COVID-19 pandemic [18,19,20,21]. We specified our ITS models based on prior literature as follows: [22]

$$Y_{it}=\beta_{0}+\beta_{1}T_{t}+\beta_{2}{Post}_{t}+\beta_{3}{Post}_{t}\times T_{t}+\beta_{4}{County}_{ct}+\beta_{5}{Hospital}_{it}+{H_{i}+\varepsilon }_{it}$$

where $Y_{it}$ is CB spending (i.e., total spending and spending in specific categories); $T_{t}$ is a linear time trend; ${Post}_{t}$ is a dummy variable measuring the post-policy periods (i.e., equals 0 for pre-policy periods and 1 for post-policy periods); and $\beta_{3}{Post}_{t}\times T_{t}$ represents the difference between pre-policy and post-policy slopes of the outcome. ${County}_{ct}$ is a vector of county characteristics (median household income and mean uninsurance rate). ${Hospital}_{it}$ is a vector of hospital characteristics (bed size and Herfindahl–Hirschman Index). Additionally, the model controls for hospital ($H_{i}$) fixed effects. We clustered the errors on hospital to correct for autocorrelation. This specification allows the pre-treatment constant $\beta_{0}$ and $\beta_{1}T_{t}$ to serve as the control. $\beta_{2}{Post}_{t}$ and $\beta_{3}{Post}_{t}\times T_{t}$ measure the changes, or the interruptions, in the pre-trend resulting from treatment.

The OHA first implemented the policy in 2020, except for the minimum spending floors that were implemented for CB spending in 2022. In the main model, the post-policy period was defined as 2020 to 2022. We also estimated models with the post-policy period defined as 2022 to examine the association between CB spending and the minimum spending floors.

To explore whether or not there was heterogeneity across hospital types, we stratified the analysis by frontier (i.e., hospitals located in a county with a population density of six or fewer people per square mile) and critical access status. We also grouped hospitals in terciles based on their pre-policy mean CB spending in 2017 and 2018. We report descriptive statistics for the full sample of hospitals, and by frontier and critical access status.

This study was approved by the Tulane University School of Public Health’s institutional review board on May 17, 2024. The need for informed consent was waived because we used deidentified secondary data. All analyses were conducted from June to December 2024 using Stata, version 18 (StataCorp LLC, College Station, TX, USA).

## 3. Results

The 56 hospitals in the sample included 49 non-frontier and seven frontier hospitals. Critical access hospitals made up 45% of the sample. Table 1 presents hospital and county-level descriptive statistics for the full sample of hospitals, by terciles of pre-policy CB spending, and by frontier and critical access status. Hospitals in the first tercile of pre-policy CB spending, frontier, and critical access hospitals are located in more concentrated markets, as indicated by larger HHI. This is expected as these hospitals are likely the only hospitals in their communities. These same hospitals are smaller in size and located in counties with lower median income and higher social vulnerability.

**Figure 1:**
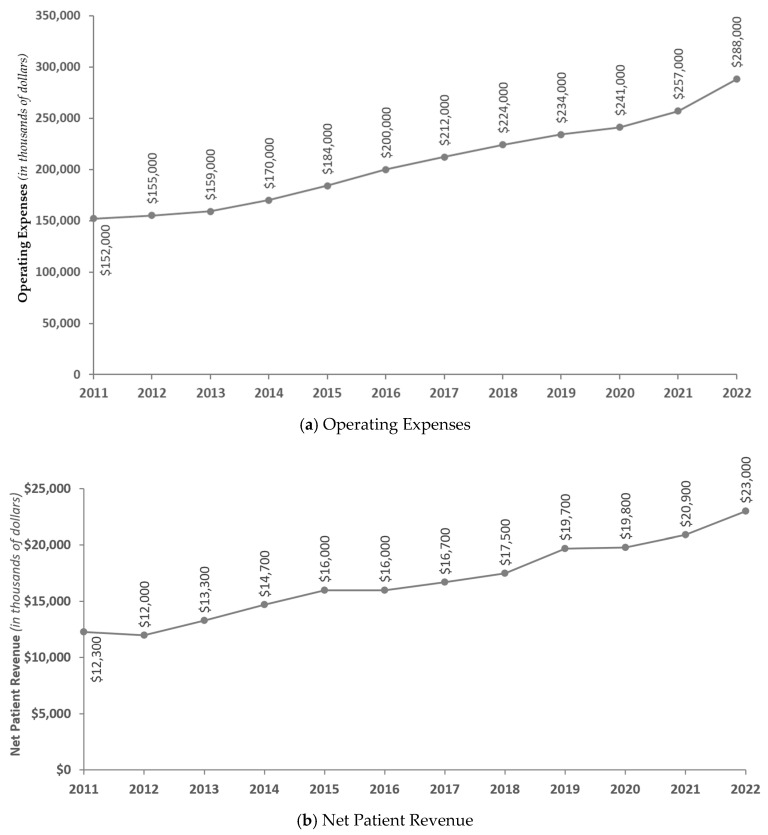
Yearly unadjusted hospital financial indicators. Note: The sample includes all nonprofit hospitals in Oregon that have complete data from 2011 to 2022 (n = 56).

Figure 1 presents the unadjusted trends of hospital operating expenses and net patient revenue for the full sample of hospitals from 2011 to 2022. Overall, both operating expenses and net patient revenue increased from 2011 to 2022.

**Figure 2:**
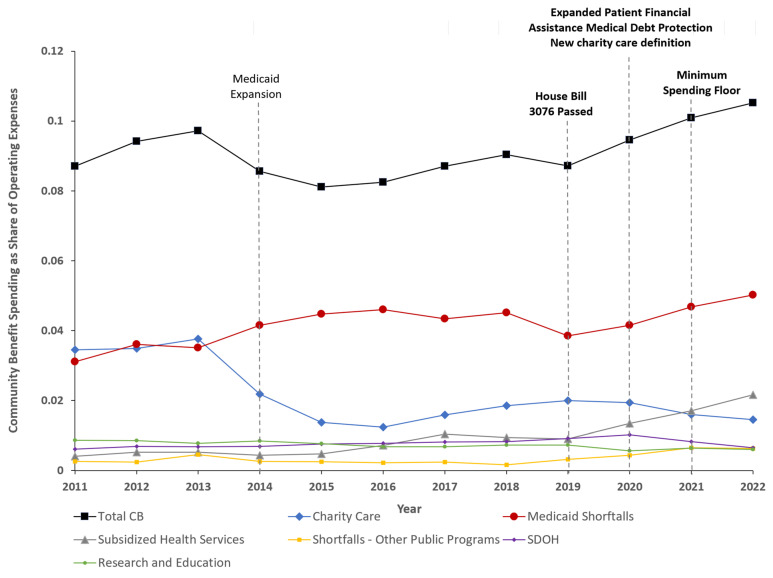
Unadjusted means of community benefit spending as share of operating expenses, 2011–2022. Abbreviations: CB, community benefit; SDOH, Social Determinants of Health. Unadjusted means for each category of CB spending from each year (2011 to 2022) are presented. The sample includes all not-for-profit hospitals in Oregon that have complete data from 2011 to 2022 (n = 56). Social Determinants of Health community benefit (CB) spending includes community health improvement and operations, cash and in-kind contributions, and community building activities. Research and education CB spending includes research and health profession education.

Figure 2 displays the unadjusted trends for each CB category for the full sample of hospitals. Overall, total CB spending increased starting in 2019 and was primarily driven by increases in shortfalls from Medicaid and other government programs, and subsidized health services. Charity care spending consistently decreased starting in 2019, while SDOH spending increased slightly in 2020 but decreased thereafter. Research and education spending remained mostly flat.

Table 2 shows the ITS analysis of CB spending as a share of operating expenses for the full sample, by terciles of pre-policy CB spending and by hospital type. In the full sample, the Oregon CB policy was not associated with changes in CB spending, except for a 0.2% decrease in SDOH spending (−0.0018; standard error (se), 0.0007; *p* < 0.05), which is equivalent to a reduction of USD 471,600 per hospital. Decreased spending in SDOH was primarily driven by hospitals in the second tercile of pre-policy CB spending and non-frontier hospitals. Among hospitals in the first tercile, the Oregon CB policy was associated with a 0.4% decrease in charity care spending (−0.0041; se, 0.0018; *p* < 0.05; USD 321K/hospital) and a 0.6% increase in subsidized health services spending (0.0063; se, 0.0032; *p* < 0.05; USD 493K/hospital). Among hospitals in the second tercile, the Oregon CB policy was associated with a 0.7% decrease in subsidized health services spending (−0.0074; se, 0.0038; *p* < 0.05; USD 2.7 million/hospital). There was no change in spending for hospitals in the third tercile. Among frontier hospitals, the Oregon policy was associated with a 2.9% increase in total CB spending (0.0292; se, 0.0124; *p* < 0.10; USD 1.1 million/hospital) and a 2.2% increase in Medicaid shortfalls (0.0220; se, 0.0108; *p* < 0.10; USD 805K/hospital), while non-frontier hospitals experienced a 0.7% decrease in Medicaid shortfall spending (−0.0068; se, 0.0034; *p* < 0.05; USD 2 million/hospital). Critical access hospitals experienced a 1.3% increase in subsidized health services spending (0.0131; se, 0.0058; *p* < 0.05; USD 899K/hospital). Appendices A to G provide full estimates for all analyses.

In separate models, we defined the post-policy period as 2022, and the findings were similar (Appendix H). We also estimated models using non-normalized spending (i.e., absolute dollars) as the outcome, and findings were consistent with the main results (Appendix I).

## 4. Discussion

In this cohort study, we found that the Oregon CB policy was not associated with changes in CB spending overall, except for a decline in SDOH spending. However, the findings were heterogenous based on hospital type and their pre-policy CB spending. For example, we found that the policy was associated with increased total CB spending for frontier hospitals. This increase was primarily driven by spending in Medicaid shortfalls, which was likely due to the continuous Medicaid enrollment protections during the public health emergency (PHE) [23,24]. In fact, Medicaid enrollment was higher in frontier counties starting in 2020 (22.4%) compared to non-frontier counties (19.8%), and this might explain the larger spending in Medicaid shortfalls by frontier hospitals. There is also indication that hospitals may be responding to the policy by changing the composition of CB spending. For instance, hospitals in the first tercile of pre-policy CB spending had lower charity care spending but higher spending on subsidized health services. Future qualitative studies can closely examine how hospitals are meeting the requirements of Oregon’s CB policy.

Amidst bipartisan policies aimed to improve transparency and accountability among NFP hospitals regarding their provision of CBs, Oregon’s multifaceted CB policy stands out [2,3]. In particular, the policy’s expanded medical debt protections for patients have the potential to address legislators’ concerns about escalating medical debt and predatory debt collection practices. Additionally, Oregon’s minimum CB spending floor formula is a potential improvement over previous policies that applied the same flat rate on net patient revenue for charity care. It is designed to avoid the “race to the bottom” phenomenon, in which previously high-spending hospitals end up spending less to meet the new spending threshold [15]. Furthermore, the formula sets an expectation that hospitals enjoying robust financial performance will spend more in CB, with the opposite being true for financially vulnerable hospitals. Consistent with the intent of the policy design, we find no evidence of a race to the bottom effect. However, given our findings of no CB spending changes after the Oregon CB policy was implemented, it is possible that the minimum CB spending amount assigned to each hospital may have been set too low. In fact, the minimum CB spending floor formula accounts for prior hospital spending in only four out of 17 categories of CB spending (i.e., charity care, shortfalls from Medicaid and other government programs, and subsidized health services), which may have led to conservative spending levels.

Although the minimum CB spending floor formula is designed to be responsive to fluctuation in hospital financial performance, it may not have accounted for the ways in which the pandemic affected hospital finances and market dynamics. On one hand, pandemic-related financial challenges could have made it harder for hospitals to increase their CB investments [25,26]. On the other hand, hospitals received billions of dollars in federal and state PHE funds as well as increased Medicaid reimbursement [27]. Given the pandemic and the phased implementation of Oregon’s CB reform, it is possible that more time is needed for hospital operations to stabilize before changes in CB spending are realized. Nonetheless, additional guidance and oversight may be necessary to spur low-spending hospitals to meaningfully increase CB spending.

Ideally, NFP hospitals allocate CB dollars based on community need, which includes both direct patient care due to uninsurance and underinsurance and non-clinical SDOH. High spending in direct patient care and low spending in SDOH may be appropriate if the demand for charity care is high in the community. However, NFP hospitals can be more proactive in allocating CB dollars by using information from the federally-mandated triennial community health needs assessment and implementation strategy to better align their CB investments with community priorities [28]. In fact, Oregon’s CB reform aspires to achieve that alignment by emphasizing CB investments in both direct patient care and SDOH. Historically, hospital spending in SDOH has not increased even as CB dollars have been freed up due to lower demand for charity care after insurance expansions from the ACA [6]. In Oregon, this pattern is apparent and points to an opportunity to refine the minimum spending floor formula to require larger spending in SDOH to alleviate disparities. Additionally, the Oregon CB policy can be amended to require hospitals to link CB spending to identified community needs. The innovative Oregon CB policy offers a regulatory framework for states seeking to enhance hospital investments in community health and accountability to the communities they serve.

### Limitations

Our analysis has limitations. We conducted an observational study and thus could only examine an association between the Oregon CB policy and the study outcomes rather than demonstrating causality. Future research should incorporate a comparison group and employ a causal inference approach, such as a difference-in-differences study design, to better assess the impact of the policy. However, this was beyond the scope of the current study, in part due to only having one year of data after the implementation of the minimum spending floors. Therefore, our findings regarding the Oregon CB policy’s impact are preliminary. As discussed above, the pandemic’s impact on hospital finances and uninsurance rates could have influenced hospital CB spending in either direction. Our models aimed to account for some of these dynamics; for example, the pandemic led to an increase in Medicaid enrollment, which may have decreased the demand for charity care [24]. Our models controlled for uninsurance rates to account for some of these dynamics. A future study could include additional years of post-implementation data. This would allow for the pandemic’s impact on hospital finances to stabilize and would minimize confounding due to pandemic shocks. Another concern is the impact of hospital market consolidation during the study period. It is possible that mergers and acquisitions may have led to decreases in CB spending, especially spending in SDOH [29]. To address this, we controlled for hospital market concentration (i.e., HHI). With ITS analysis, there is a risk of biases from auto-correlation and seasonality [30]. To correct for auto-correlation, we clustered standard errors on hospitals, which may minimize bias from this source. To address potential bias from seasonality, we included a linear time trend. Additionally, hospitals’ operating expenses increased faster than CB spending since COVID-19 began and could have artificially attenuated the policy impact. To address this concern, we also used raw dollar CB spending as the outcome (see Appendix C). Finally, we had to drop four hospitals due to missing data. It is unclear how the exclusion of these hospitals would have impacted our findings. For instance, if all excluded hospitals were high-spending hospitals in terms of high CB spending, our point estimates could have been larger in magnitude and standard errors would have been lower. However, if excluded hospitals were low-spending hospitals, our point estimates could have been smaller in magnitude. Despite these limitations, our study leveraged comprehensive CB data and rigorous analyses to examine the impact of the novel Oregon CB policy.

## 5. Conclusions

It is still too early to draw firm conclusions from our findings. Nevertheless, our findings point towards few changes in CB spending, except for a concerning decline in SDOH spending. Importantly, we found no evidence of a “race to the bottom”, but opportunities exist to fine-tune the minimum spending formula to help Oregon boost NFP hospital CB spending. Proactive CB spending is a cornerstone of the Oregon CB policy, and the state’s CB program is primed for policy improvements that can increase spending in categories such as charity care and subsidized health services to help alleviate the increasing burden of medical debt and its financial implications for individuals across the US.

Given the complex dynamics of hospital finances during the pandemic and the implementation of Oregon’s forward-looking CB policy, it is possible that more time is needed for hospital operations to stabilize before changes in CB spending are realized. As the economy stabilizes and hospitals experience less uncertainty, it will be important to monitor the impact of Oregon’s CB policy on hospital CB investments. The formula was developed with the flexibility to accommodate changes that can help Oregon reach its goal of addressing unmet health needs and health inequities.

## Figures and Tables

**Table 1:** County characteristics by hospital type.

	All Hospitals (n = 56)	Tercile of Pre-Policy Community Benefit Spending			Frontier (n = 7)	Non-Frontier (n = 49)	Critical Access Hospitals (n = 25)
		1st Tercile (n = 19)	2nd Tercile (n = 19)	3rd Tercile (n = 18)			
**County and Hospital Characteristics (sd)**							
HHI	0.69 (0.27)	0.79 (0.22)	0.62 (0.25)	0.66 (0.31)	0.99 (0.11)	0.65 (0.26)	0.81 (0.22)
Beds	110 (138)	37 (35)	156 (149)	138 (163)	26 (10)	122 (144)	22 (5)
Uninsurance (%)	10.0 (3.8)	10.0 (3.8)	10.4 (3.8)	9.6 (3.8)	10.2 (4.0)	10.0 (3.8)	10.35 (3.85)
Median Income (USD)	52,575 (11,321)	47,685 (8731)	56,005 (11,665)	54,117 (11,644)	42,887 (6284)	53,959 (11,206)	47,348 (7220)
SVM	−0.10 (0.59)	0.20 (0.40)	−0.27 (0.63)	−0.25 (0.61)	0.56 (0.26)	−0.20 (0.57)	0.24 (0.29)

Abbreviations: sd, standard deviation; HHI, Herfindahl–Hirschman Index; SVM, social vulnerability metric. County characteristics are based on pre-policy data (2019). Larger values of the social vulnerability metric indicate higher levels of social vulnerability. The sample includes all nonprofit hospitals in Oregon that have complete data from 2011 to 2022 (n = 56).

**Table 2:** Interrupted time-series estimates of the association between Oregon’s community benefit policy and community benefit spending as a share of operating expenses.

All Hospitals (n = 56)							
Total Community Benefit	Direct Patient Care	SDOH	Research and Education	Charity Care	Medicaid Shortfalls	Other Govt Program Shortfalls	Subsidized Health Services
−0.0009	0.0010	−0.0018 **	−0.0002	−0.0008	−0.0018	0.0006	0.0031
(0.0050)	(0.0048)	(0.0007)	(0.0004)	(0.0014)	(0.0037)	(0.0006)	(0.0030)
**Hospitals in 1st Tercile of Pre-Policy Community Benefit Spending** (n = 19)							
0.0026	0.0028	0.00001	−0.0002	−0.0041 **	−0.0005	0.0011	0.0063 **
(0.0077)	(0.0080)	(0.0013)	(0.0006)	(0.0018)	(0.0083)	(0.0008)	(0.0032)
**Hospitals in 2nd Tercile of Pre-Policy Community Benefit Spending** (n = 19)							
−0.0083	−0.0051	−0.0041 ***	0.0009	0.0016	−0.0002	0.0010	−0.0074 ^+^
(0.0053)	(0.0050)	(0.0010)	(0.0006)	(0.0024)	(0.0042)	(0.0012)	(0.0038)
**Hospitals in 3rd Tercile of Pre-Policy Community Benefit Spending** (n = 18)							
−0.0006	0.0033	−0.0009	−0.003	0.0027	−0.0055	−0.0006	0.0068
(0.0112)	(0.0105)	(0.0013)	(0.0025)	(0.0037)	(0.0062)	(0.0013)	(0.0076)
**Frontier Hospitals** (n = 7)							
0.0292 ^+^	0.0286 ^+^	0.0009	−0.0002	−0.0008	0.0220 ^+^	0.002	0.0054
(0.0124)	(0.0118)	(0.0022)	(0.0006)	(0.0023)	(0.0108)	(0.0011)	(0.0035)
**Non-Frontier Hospitals** (n = 49)							
−0.0070	−0.0050	−0.0017 **	−0.0003	0.0004	−0.0068 **	0.00004	0.0013
(0.0045)	(0.0044)	(0.0008)	(0.0006)	(0.0017)	(0.0034)	(0.0008)	(0.0036)
**Critical Access Hospitals** (n = 25)							
0.0075	0.0078	−0.0002	−0.0001	−0.0030	−0.0027	0.0004	0.0131 **
(0.0093)	(0.0091)	(0.0009)	(0.0005)	(0.0020)	(0.0072)	(0.0012)	(0.0058)

Abbreviations: SDOH, Social Determinants of Health; Govt, government. The sample includes all nonprofit hospitals in Oregon that have complete data from 2011 to 2022 (n = 56). Hospitals were stratified by terciles of pre-policy community benefit (CB) spending (mean of 2017 and 2018). Direct patient care community benefit (CB) spending includes direct patient care charity care, shortfalls from Medicaid and other government programs, and subsidized health services. Social Determinants of Health community benefit (CB) spending includes community health improvement and operations, cash and in-kind contributions, and community building activities. Research and Education CB spending includes research and health profession education. Results of regression analyses are presented, adjusted for hospital bed size and county-level characteristics including uninsurance rate, median household income, and Herfindahl–Hirschman Index. All models control for hospital fixed effects. Standard errors are provided in parentheses and are clustered on hospitals. Bolded coefficients are statistically significant. *** *p* < 0.01, ** *p* < 0.05, ^+^ *p* < 0.1.

## Data Availability

Tatiane Santos had full access to all the data in the study and takes responsibility for the integrity of the data and the accuracy of the data analysis. Data can be requested via an email to Tatiane Santos.

## Abbreviations

The following abbreviations are used in this manuscript:

CB	Community benefit
NFP	Not-for-profit
ACA	Patient Protection and Affordable Care Act
OHA	Oregon Health Authority
SDOH	Social Determinants of Health
ITS	Interrupted time series
HHI	Herfindahl–Hirschman Index

## Appendix A

Interrupted Time-Series Estimates of the Association between Oregon’s Minimum Spending Floor and Community Benefit Spending as a Share of Operating Expenses: Full Sample.

	**Total Community Benefit**	**Direct Patient Care**	**SDOH**	**Research and Education**	**Charity Care**	**Medicaid Shortfalls**	**Other Govt Program Shortfalls**	**Subsidized Health Services**
**Post-policy slope change**	−0.000905	0.00103	−0.00175 **	−0.000189	−0.000834	−0.00177	0.000555	0.00309
Post(t) × T(t)	(0.00495)	(0.00484)	(0.000678)	(0.000445)	(0.00133)	(0.00391)	(0.000621)	(0.00310)
**Linear time trend (years)**	0.00597	0.00567	−0.000121	0.000424	−0.00156	0.00582 *	0.000449	0.000962
T(t)	(0.00387)	(0.00383)	(0.000460)	(0.000532)	(0.00127)	(0.00307)	(0.000579)	(0.00228)
**Intervention**	0.00795	−0.00315	0.0172 ***	0.00119	0.00964	0.0120	−0.00288	−0.0219
Post(t)	(0.00607)	(0.0404)	(0.00622)	(0.00373)	(0.0112)	(0.0326)	(0.00540)	(0.0264)
Median Income (USD)	−1.31 × 10^−6^ *	−1.10 × 10^−6^	2.25 × 10^−8^	−2.29 × 10^−7^	8.67 × 10^−7^ ***	−1.22 × 10^−6^ **	−5.02 × 10^−8^	−6.98 × 10^−7^ **
	(7.07 × 10^−7^)	(6.84 × 10^−7^)	(1.49 × 10^−7^)	(2.28 × 10^−7^)	(2.59 × 10^−7^)	(5.46 × 10^−7^)	(1.18 × 10^−7^)	(3.22 × 10^−7^)
Uninsured (%)	0.00249	0.00266	−0.000270	0.000105	0.00123 *	0.00184	0.000338	−0.000748
	(0.00232)	(0.00223)	(0.000353)	(0.000211)	(0.000648)	(0.00167)	(0.000407)	(0.00153)
Herfindahl–Hirschman Index	−0.00408	−0.00365	0.00206	−0.00249	−0.00671	0.0118	0.00460	−0.0133
	(0.0274)	(0.0240)	(0.00209)	(0.00318)	(0.00454)	(0.0122)	(0.00456)	(0.0128)
Number of hospital beds	0.000124 *	0.000140 **	7.74 × 10^−6^	−2.38 × 10^−5^	−2.25 × 10^−5^	0.000156	3.05 × 10^−6^	3.37 × 10^−6^
	(6.37 × 10^−5^)	(6.98 × 10^−5^)	(2.03 × 10^−5^)	(2.13 × 10^−5^)	(4.20 × 10^−5^)	(9.47 × 10^−5^)	(1.20 × 10^−5^)	(3.49 × 10^−5^)
Constant	0.133 ***	0.0568	0.00807	0.0201 *	−0.0231	0.0290	−0.00427	0.0551
	(0.0476)	(0.0511)	(0.0100)	(0.0107)	(0.0157)	(0.0358)	(0.00804)	(0.0361)
Observations	672	672	672	672	672	672	672	672
R-squared	0.065	0.082	0.032	0.042	0.221	0.045	0.073	0.150
SOURCE Authors’ analyses of audited hospital financial data and community benefit spending from the Oregon Health Authority Hospital Reporting Program and Agency for Healthcare Research and Quality Social Determinants of Health Database. NOTES The sample includes all nonprofit hospitals in Oregon that have complete data from 2011 to 2022 (n = 56). Results of regression analyses are presented, adjusted for hospital bed size and county-level characteristics including uninsurance rate, median household income, and Herfindahl–Hirschman Index. Intervention Post(t) is a dummy variable that equals 1 for year ≥ 2020, and 0 otherwise. All models control for hospital fixed effects. Standard errors are clustered on hospitals. *** *p* < 0.01, ** *p* < 0.05, * *p* < 0.1.								

## Appendix B

Interrupted Time–Series Estimates of the Association between Oregon’s Minimum Spending Floor and Community Benefit Spending as a Share of Operating Expenses: Hospitals in 1st Tercile of Pre-Policy Community Benefit Spending.

	**Total Community Benefit**	**Direct Patient Care**	**SDOH**	**Research and Education**	**Charity Care**	**Medicaid Shortfalls**	**Other Govt Program Shortfalls**	**Subsidized Health Services**
**Post-policy slope change**	−0.00833	−0.00508	−0.00412 ***	0.000862	0.00156	−0.000201	0.000972	−0.00741 *
Post(t) × T(t)	(0.00512)	(0.00502)	(0.00102)	(0.000616)	(0.00231)	(0.00410)	(0.00111)	(0.00401)
**Linear time trend (years)**	0.00518	0.00549	0.000556	−0.000864	−0.00402 *	0.00298	0.000131	0.00640 *
T(t)	(0.00350)	(0.00372)	(0.000791)	(0.000577)	(0.00209)	(0.00298)	(0.00103)	(0.00356)
**Intervention**	0.0812 *	0.0494	0.0391 ***	−0.00731	−0.0147	0.00135	−0.00819	0.0710 *
Post(t)	(0.0451)	(0.0438)	(0.00930)	(0.00517)	(0.0190)	(0.0358)	(0.00921)	(0.0368)
Median Income (USD)	−8.04 × 10^−7^	−9.32 × 10^−7^	4.06 × 10^−8^	8.67 × 10^−7^	1.40 × 10^−6^ ***	−1.39 × 10^−6^ **	5.44 × 10^−8^	−1.00 × 10^−7^ ***
	(6.88 × 10^−7^)	(7.06 × 10^−7^)	(2.13 × 10^−7^)	(9.72 × 10^−8^)	(4.11 × 10^−7^)	(5.83 × 10^−7^)	(2.30 × 10^−7^)	(3.47 × 10^−7^)
Uninsured (%)	0.00300	0.00312	0.000189	−0.000312	0.000509	0.000152	0.000238	0.00222
	(0.00213)	(0.00223)	(0.000292)	(0.000224)	(0.000922)	(0.00165)	(0.000322)	(0.00190)
Herfindahl–Hirschman Index	−0.0456	−0.0552	0.0122	−0.00258	0.00562	−0.0504	−0.0306	0.0203
	(0.113)	(0.110)	(0.0113)	(0.00740)	(0.0253)	(0.0811)	(0.0196)	(0.0264)
Number of hospital beds	0.000188 **	0.000187 **	1.09 × 10^−5^	−9.64 × 10^−6^	6.99 × 10^−5^ **	6.02 × 10^−5^	1.43 × 10^−5^	4.27 × 10^−5^
	(8.38 × 10^−5^)	(8.84 × 10^−5^)	(2.12 × 10^−5^)	(1.38 × 10^−5^)	(2.75 × 10^−5^)	(5.56 × 10^−5^)	(1.86 × 10^−5^)	(4.44 × 10^−5^)
Constant	0.0723	0.0686	−0.00871	0.0124 *	−0.0555 *	0.125 **	0.0156	−0.0167
	(0.0650)	(0.0648)	(0.0151)	(0.00605)	(0.0285)	(0.0500)	(0.0118)	(0.0382)
Observations	228	228	228	228	228	228	228	228
R−squared	0.059	0.066	0.195	0.086	0.298	0.066	0.079	0.211
SOURCE Authors’ analyses of audited hospital financial data and community benefit spending from the Oregon Health Authority Hospital Reporting Program and Agency for Healthcare Research and Quality Social Determinants of Health Database. NOTES The sample includes all nonprofit hospitals in Oregon that have complete data from 2011 to 2022 (n = 56). Results of regression analyses are presented, adjusted for hospital bed size and county-level characteristics including uninsurance rate, median household income, and Herfindahl–Hirschman Index. Intervention Post(t) is a dummy variable that equals 1 for year ≥ 2020, and 0 otherwise. Hospitals were stratified by terciles of pre-policy community benefit (CB) spending (mean of 2017 and 2018). All models control for hospital fixed effects. Standard errors are clustered on hospitals. *** *p* < 0.01, ** *p* < 0.05, * *p* < 0.1.								

## Appendix C

Interrupted Time-Series Estimates of the Association between Oregon’s Minimum Spending Floor and Community Benefit Spending as a Share of Operating Expenses: Hospitals in 2nd Tercile of Pre-Policy Community Benefit Spending.

	**Total Community Benefit**	**Direct Patient Care**	**SDOH**	**Research and Education**	**Charity Care**	**Medicaid Shortfalls**	**Other Govt Program Shortfalls**	**Subsidized Health Services**
**Post-policy slope change**	−0.00833	−0.00508	−0.00412 ***	0.000862	0.00156	−0.000201	0.000972	−0.00741 *
Post(t) × T(t)	(0.00512)	(0.00502)	(0.00102)	(0.000616)	(0.00231)	(0.00410)	(0.00111)	(0.00401)
**Linear time trend (years)**	0.00518	0.00549	0.000556	−0.000864	−0.00402 *	0.00298	0.000131	0.00640 *
T(t)	(0.00350)	(0.00372)	(0.000791)	(0.000577)	(0.00209)	(0.00298)	(0.00103)	(0.00356)
**Intervention**	0.0812 *	0.0494	0.0391 ***	−0.00731	−0.0147	0.00135	−0.00819	0.0710 *
Post(t)	(0.0451)	(0.0438)	(0.00930)	(0.00517)	(0.0190)	(0.0358)	(0.00921)	(0.0368)
Median Income (USD)	−8.04 × 10^−7^	−9.32 × 10^−7^	4.06 × 10^−8^	8.67 × 10^−8^	1.40 × 10^−6^ ***	−1.39 × 10^−6^ **	5.44 × 10^−8^	−1.00 × 10^−6^ ***
	(6.88 × 10^−7^)	(7.06 × 10^−7^)	(2.13 × 10^−7^)	(9.72 × 10^−8^)	(4.11 × 10^−7^)	(5.83 × 10^−7^)	(2.30 × 10^−7^)	(3.47 × 10^−7^)
Uninsured (%)	0.00300	0.00312	0.000189	−0.000312	0.000509	0.000152	0.000238	0.00222
	(0.00213)	(0.00223)	(0.000292)	(0.000224)	(0.000922)	(0.00165)	(0.000322)	(0.00190)
Herfindahl–Hirschman Index	−0.0456	−0.0552	0.0122	−0.00258	0.00562	−0.0504	−0.0306	0.0203
	(0.113)	(0.110)	(0.0113)	(0.00740)	(0.0253)	(0.0811)	(0.0196)	(0.0264)
Number of hospital beds	0.000188 **	0.000187 **	1.09 × 10^−5^	−9.64 × 10^−6^	6.99 × 10^−5^ **	6.02 × 10^−5^	1.43 × 10^−5^	4.27 × 10^−5^
	(8.38 × 10^−5^)	(8.84 × 10^−5^)	(2.12 × 10^−5^)	(1.38 × 10^−5^)	(2.75 × 10^−5^)	(5.56 × 10^−5^)	(1.86 × 10^−5^)	(4.44 × 10^−5^)
Constant	0.0723	0.0686	−0.00871	0.0124 *	−0.0555 *	0.125 **	0.0156	−0.0167
	(0.0650)	(0.0648)	(0.0151)	(0.00605)	(0.0285)	(0.0500)	(0.0118)	(0.0382)
Observations	228	228	228	228	228	228	228	228
R−squared	0.059	0.066	0.195	0.086	0.298	0.066	0.079	0.211
SOURCE Authors’ analyses of audited hospital financial data and community benefit spending from the Oregon Health Authority Hospital Reporting Program and Agency for Healthcare Research and Quality Social Determinants of Health Database. NOTES The sample includes all nonprofit hospitals in Oregon that have complete data from 2011 to 2022 (n = 56). Results of regression analyses are presented, adjusted for hospital bed size and county-level characteristics including uninsurance rate, median household income, and Herfindahl–Hirschman Index. Intervention Post(t) is a dummy variable that equals 1 for year ≥ 2020, and 0 otherwise. Hospitals were stratified by terciles of pre-policy community benefit (CB) spending (mean of 2017 and 2018). All models control for hospital fixed effects. Standard errors are clustered on hospitals. *** *p* < 0.01, ** *p* < 0.05, * *p* < 0.1.								

## Appendix D

Interrupted Time-Series Estimates of the Association between Oregon’s Minimum Spending Floor and Community Benefit Spending as a Share of Operating Expenses: Hospitals in 3rd Tercile of Pre-Policy Community Benefit Spending.

	**Total Community Benefit**	**Direct Patient Care**	**SDOH**	**Research and Education**	**Charity Care**	**Medicaid Shortfalls**	**Other Govt Program Shortfalls**	**Subsidized Health Services**
**Post-policy slope change**	−0.000586	0.00332	−0.000905	−0.00300	0.00271	−0.00553	−0.000619	0.00676
Post(t) × T(t)	(0.0111)	(0.0105)	(0.00126)	(0.00233)	(0.00351)	(0.00619)	(0.00129)	(0.00717)
**Linear time trend (years)**	0.0138	0.0110	−0.000419	0.00321	−0.00540	0.0114 *	0.00226 *	0.00274
T(t)	(0.00932)	(0.00871)	(0.000814)	(0.00276)	(0.00348)	(0.00550)	(0.00122)	(0.00579)
**Intervention**	−0.00307	−0.0383	0.0103	0.0249	−0.0181	0.0316	0.00541	−0.0572
Post(t)	(0.0918)	(0.0867)	(0.0123)	(0.0199)	(0.0303)	(0.0502)	(0.0109)	(0.0603)
Median Income (USD)	−2.47 × 10^−6^	−1.43 × 10^−6^	1.56 × 10^−9^	−1.04 × 10^−6^	1.83 × 10^−6^ **	−1.16 × 10^−6^	−1.47 × 10^−7^	−1.95 × 10^−6^ *
	(2.05 × 10^−6^)	(1.86 × 10^−6^)	(1.87 × 10^−7^)	(9.25 × 10^−7^)	(6.87 × 10^−7^)	(1.11 × 10^−6^)	(2.40 × 10^−7^)	(1.10 × 10^−6^)
Uninsured (%)	0.00409	0.00355	−0.000403	0.000945	0.000486	0.00370	0.00152	−0.00215
	(0.00588)	(0.00562)	(0.000523)	(0.00104)	(0.00177)	(0.00362)	(0.00104)	(0.00362)
Herfindahl–Hirschman Index	−0.195	−0.171	−0.00311	−0.0208	−0.0561 **	0.00916	−0.00665	−0.118 **
	(0.124)	(0.105)	(0.0109)	(0.0167)	(0.0238)	(0.0760)	(0.00892)	(0.0466)
Number of hospital beds	0.000125	0.000115	2.05 × 10^−5^	−1.09 × 10^−5^	−0.000124 *	0.000238 **	2.59 × 10^−5^	−2.45 × 10^−5^
	(0.000142)	(0.000148)	(4.11 × 10^−5^)	(3.71 × 10^−5^)	(5.93 × 10^−5^)	(0.000107)	(2.19 × 10^−5^)	(4.99 × 10^−5^)
Constant	0.246	0.177	0.0125	0.0568	0.000458	−0.00965	−0.0168	0.203 **
	(0.154)	(0.137)	(0.0146)	(0.0355)	(0.0327)	(0.0819)	(0.0202)	(0.0875)
Observations	228	228	228	228	228	228	228	228
R−squared	0.125	0.140	0.055	0.120	0.279	0.100	0.105	0.263
SOURCE Authors’ analyses of audited hospital financial data and community benefit spending from the Oregon Health Authority Hospital Reporting Program and Agency for Healthcare Research and Quality Social Determinants of Health Database. NOTES The sample includes all nonprofit hospitals in Oregon that have complete data from 2011 to 2022 (n = 56). Direct patient care community benefit (CB) spending includes direct patient care charity care, shortfalls from Medicaid and other gov-ernment programs, and subsidized health services. Social Determinants of Health community benefit (CB) spending includes community health improvement and operations, cash and in-kind contributions, and community building activities. Research and Education CB spending includes research and health profession education. Results of regression analyses are presented, adjusted for hospital bed size and county-level characteristics including uninsurance rate, median household income, and Herfindahl–Hirschman Index. Intervention Post(t) is a dummy variable that equals 1 for year ≥ 2020, and 0 otherwise. Hospitals were stratified by terciles of pre-policy community benefit (CB) spending (mean of 2017 and 2018). All models control for hospital fixed effects. Standard errors are clustered on hospitals. ** *p* < 0.05, * *p* < 0.1.								

## Appendix E

Interrupted Time-Series Estimates of the Association between Oregon’s Minimum Spending Floor and Community Benefit Spending as a Share of Operating Expenses: Frontier Hospitals.

	**Total Community Benefit**	**Direct Patient Care**	**SDOH**	**Research and Education**	**Charity Care**	**Medicaid Shortfalls**	**Other Govt Program Shortfalls**	**Subsidized Health Services**
**Post-policy slope change**	0.0292 *	0.0286 *	0.000882	−0.000215	−0.000815	0.0220 *	0.00200	0.00542
Post(t) × T(t)	(0.0124)	(0.0118)	(0.00220)	(0.000604)	(0.00230)	(0.0108)	(0.00113)	(0.00348)
**Linear time trend (years)**	−0.00975	−0.00975 *	−0.000371	0.000369	0.000842	−0.00889 **	6.68 × 10^−5^	−0.00177
T(t)	(0.00567)	(0.00494)	(0.00115)	(0.000383)	(0.00209)	(0.00342)	(0.000374)	(0.00143)
**Intervention**	−0.237 *	−0.229 *	−0.00948	0.00127	0.00849	−0.172	−0.0190	−0.0459
Post(t)	(0.117)	(0.112)	(0.0208)	(0.00532)	(0.0173)	(0.108)	(0.0116)	(0.0298)
Median Income (USD)	−1.81 × 10^−7^	−1.01 × 10^−6^	1.01 × 10^−6^	−1.82 × 10^−7^	2.26 × 10^−7^	−3.24 × 10^−7^	9.51 × 10^−8^	−1.01 × 10^−6^
	(3.19 × 10^−6^)	(2.63 × 10^−6^)	(1.09 × 10^−6^)	(2.22 × 10^−7^)	(6.92 × 10^−7^)	(2.44 × 10^−6^)	(5.00 × 10^−7^)	(6.42 × 10^−7^)
Uninsured (%)	−0.00665 *	−0.00630 *	−0.000573	0.000225	0.00146	−0.00590 **	0.000104	−0.00196
	(0.00296)	(0.00258)	(0.000991)	(0.000262)	(0.00123)	(0.00167)	(0.000293)	(0.00161)
Herfindahl–Hirschman Index	0.0202 *	0.0150	0.00587	−0.000647	−0.00256	0.0374 ***	0.000910	−0.0208 ***
	(0.00881)	(0.00922)	(0.00365)	(0.000734)	(0.00365)	(0.00737)	(0.00208)	(0.00319)
Number of hospital beds	−0.000324	0.000239	−0.000668	0.000105	3.57 × 10^−5^	0.000404	0.000251	−0.000452
	(0.00175)	(0.00157)	(0.000451)	(0.000101)	(0.000237)	(0.00152)	(0.000496)	(0.000417)
Constant	0.176	0.188	−0.0151	0.00264	−0.0158	0.106	−0.0120	0.110 *
	(0.130)	(0.115)	(0.0309)	(0.00448)	(0.0378)	(0.0881)	(0.0217)	(0.0496)
Observations	84	84	84	84	84	84	84	84
R−squared	0.413	0.418	0.167	0.152	0.180	0.397	0.144	0.279
SOURCE Authors’ analyses of audited hospital financial data and community benefit spending from the Oregon Health Authority Hospital Reporting Program and Agency for Healthcare Research and Quality Social Determinants of Health Database. NOTES The sample includes all nonprofit hospitals in Oregon that have complete data from 2011 to 2022 (n = 56). Direct patient care community benefit (CB) spending includes direct patient care charity care, shortfalls from Medicaid and other government programs, and subsidized health services. Social Determinants of Health community benefit (CB) spending includes community health improvement and operations, cash and in-kind contributions, and community building activities. Research and Education CB spending includes research and health profession education. Results of regression analyses are presented, adjusted for hospital bed size and county-level characteristics including uninsurance rate, median household income, and Herfindahl–Hirschman Index. Intervention Post(t) is a dummy variable that equals 1 for year ≥ 2020, and 0 otherwise. All models control for hospital fixed effects. Standard errors are clustered on hospitals. *** *p* < 0.01, ** *p* < 0.05, * *p* < 0.1.								

## Appendix F

Interrupted Time-Series Estimates of the Association between Oregon’s Minimum Spending Floor and Community Benefit Spending as a Share of Operating Expenses: Non-Frontier Hospitals.

	**Total Community Benefit**	**Direct Patient Care**	**SDOH**	**Research and Education**	**Charity Care**	**Medicaid Shortfalls**	**Other Govt Program Shortfalls**	**Subsidized Health Services**
**Post-policy slope change**	−0.00699	−0.00499	−0.00168 **	−0.000321	0.000457	−0.00675 **	4.51 × 10^−5^	0.00126
Post(t) × T(t)	(0.00452)	(0.00436)	(0.000823)	(0.000650)	(0.00169)	(0.00335)	(0.000759)	(0.00362)
**Linear time trend (years)**	0.0100 ***	0.00989 ***	−0.000396	0.000534	−0.00320 *	0.00939 ***	0.000756	0.00295
T(t)	(0.00364)	(0.00344)	(0.000630)	(0.000783)	(0.00162)	(0.00280)	(0.000715)	(0.00279)
**Intervention**	0.0646 *	0.0459	0.0161 **	0.00253	−0.00175	0.0504 *	0.00197	−0.00474
Post(t)	(0.0375)	(0.0365)	(0.00764)	(0.00556)	(0.0141)	(0.0279)	(0.00645)	(0.0307)
Median Income (USD)	−1.34 × 10^−6^ *	−1.23 × 10^−6^ *	1.64 × 10^−7^	−2.68 × 10^−7^	1.25 × 10^−6^ ***	−1.24 × 10^−6^ **	−1.08 × 10^−7^	−1.14 × 10^−6^ ***
	(7.94 × 10^−7^)	(7.24 × 10^−7^)	(1.61 × 10^−7^)	(2.71 × 10^−7^)	(2.87 × 10^−7^)	(5.44 × 10^−7^)	(1.39 × 10^−7^)	(4.01 × 10^−7^)
Uninsured (%)	0.00500 **	0.00504 **	−0.000146	0.000113	0.000788	0.00393 **	0.000464	−0.000144
	(0.00237)	(0.00226)	(0.000276)	(0.000298)	(0.000809)	(0.00161)	(0.000498)	(0.00180)
Herfindahl–Hirschman Index	−0.0240	−0.0147	−0.00109	−0.00828	−0.00345	−0.00695	0.00954	−0.0138
	(0.0884)	(0.0785)	(0.00492)	(0.00807)	(0.0155)	(0.0325)	(0.0129)	(0.0415)
Number of hospital beds	0.000152 **	0.000162 **	1.43 × 10^−5^	−2.36 × 10^−5^	−2.29 × 10^−5^	0.000180 *	2.18 × 10^−6^	2.33 × 16^−5^
	(6.16 × 10^−5^)	(6.93 × 10^−5^ 5)	(1.87 × 10^−5^)	(2.10 × 10^−5^)	(4.58 × 10^−5^)	(9.16 × 10^−5^)	(1.32 × 10^−5^)	(3.37 × 10^−5^)
Constant	0.0522	0.0237	0.00197	0.0265 *	−0.0320	2.10 × 10^−5^	−0.00675	0.0625
	(0.0838)	(0.0758)	(0.00867)	(0.0132)	(0.0204)	(0.0412)	(0.0104)	(0.0489)
Observations	588	588	588	588	588	588	588	588
R−squared	0.053	0.070	0.045	0.043	0.252	0.044	0.075	0.171
SOURCE Authors’ analyses of audited hospital financial data and community benefit spending from the Oregon Health Authority Hospital Reporting Program and Agency for Healthcare Research and Quality Social Determinants of Health Database. NOTES The sample includes all nonprofit hospitals in Oregon that have complete data from 2011 to 2022 (n = 56). Direct patient care community benefit (CB) spending includes direct patient care charity care, shortfalls from Medicaid and other government programs, and subsidized health services. Social Determinants of Health community benefit (CB) spending includes community health improvement and operations, cash and in-kind contributions, and community building activities. Research and Education CB spending includes research and health profession education. Results of regression analyses are presented, adjusted for hospital bed size and county-level characteristics including uninsurance rate, median household income, and Herfindahl–Hirschman Index. Intervention Post(t) is a dummy variable that equals 1 for year ≥ 2020, and 0 otherwise. All models control for hospital fixed effects. Standard errors are clustered on hospitals. *** *p* < 0.01, ** *p* < 0.05, * *p* < 0.1.								

## Appendix G

Interrupted Time-Series Estimates of the Association between Oregon’s Minimum Spending Floor and Community Benefit Spending as a Share of Operating Expenses: Critical Access Hospitals

	**Total Community Benefit**	**Direct Patient Care**	**SDOH**	**Research and Education**	**Charity Care**	**Medicaid Shortfalls**	**Other Govt Program Shortfalls**	**Subsidized Health Services**
**Post-policy slope change**	0.00752	0.00785	−0.000231	−0.000104	−0.00301	−0.00267	0.000439	0.0131 **
Post(t) × T(t)	(0.00932)	(0.00907)	(0.000941)	(0.000474)	(0.00200)	(0.00720)	(0.00121)	(0.00575)
**Linear time trend (years)**	0.00102	0.00153	−0.000752	0.000237	0.000140	0.00665	0.000368	−0.00562
T(t)	(0.00693)	(0.00679)	(0.000737)	(0.000415)	(0.00180)	(0.00545)	(0.00111)	(0.00367)
**Intervention**	−0.0473	−0.0519	0.00388	0.000729	0.0258	0.0282	−0.00213	−0.104 **
Post(t)	(0.0784)	(0.0766)	(0.00785)	(0.00372)	(0.0169)	(0.0602)	(0.0111)	(0.0493)
Median Income (USD)	−2.20 × 10^−6^ **	−2.13 × 10^−6^ **	−2.23 × 10^−8^	−4.42 × 10^−8^	8.36 × 10^−7^ *	−2.44 × 10^−6^ ***	2.08 × 10^−7^	−7.36 × 10^−7^
	(9.66 × 10^−7^)	(1.02 × 10^−6^)	(1.34 × 10^−7^)	(7.24 × 10^−8^)	(4.79 × 10^−7^)	(8.16 × 10^−7^)	(2.49 × 10^−7^)	(4.62 × 10^−7^)
Uninsured (%)	−0.000269	0.000303	−0.000727	0.000155	0.00165	0.00270	0.000563	−0.00461 *
	(0.00434)	(0.00410)	(0.000666)	(0.000237)	(0.00106)	(0.00285)	(0.000777)	(0.00260)
Herfindahl–Hirschman Index	0.0218	0.0190	0.00301	−0.000242	−0.00175	0.0150	0.00603	−0.000264
	(0.0155)	(0.0158)	(0.00231)	(0.000644)	(0.00565)	(0.00909)	(0.00639)	(0.0102)
Number of hospital beds	0.000273	0.000426	−0.000137	−1.64 × 10^−5^	0.000271 **	0.000539	−0.000116	−0.000269
	(0.000730)	(0.000649)	(0.000129)	(2.53× 10^−5^)	(0.000111)	(0.000366)	(9.26 × 10^−5^)	(0.000301)
Constant	0.144	0.119	0.0220 *	0.00217	−0.0432	0.0488	−0.0182	0.132 **
	(0.0865)	(0.0847)	(0.0108)	(0.00659)	(0.0280)	(0.0545)	(0.0125)	(0.0594)
Observations	300	300	300	300	300	300	300	300
R−squared	0.097	0.113	0.024	0.014	0.192	0.090	0.063	0.201
SOURCE Authors’ analyses of audited hospital financial data and community benefit spending from the Oregon Health Authority Hospital Reporting Program and Agency for Healthcare Research and Quality Social Determinants of Health Database. NOTES The sample includes all nonprofit hospitals in Oregon that have complete data from 2011 to 2022 (n = 56). Direct patient care community benefit (CB) spending includes direct patient care charity care, shortfalls from Medicaid and other government programs, and subsidized health services. Social Determinants of Health community benefit (CB) spending includes community health improvement and operations, cash and in-kind contributions, and community building activities. Research and Education CB spending includes research and health profession education. Results of regression analyses are presented, adjusted for hospital bed size and county-level characteristics including uninsurance rate, median household income, and Herfindahl–Hirschman Index. Intervention Post(t) is a dummy variable that equals 1 for year ≥ 2020, and 0 otherwise. All models control for hospital fixed effects. Standard errors are clustered on hospitals. *** *p* < 0.01, ** *p* < 0.05, * *p* < 0.1.								

## Appendix H

Interrupted Time-Series Estimates of the Association between Oregon’s Minimum Spending Floor and Community Benefit Spending as a Share of Operating Expenses: Post-Policy Period Defined as 2022.

	**Total Community Benefit**	**Direct Patient Care**	**SDOH**	**Research and Education**	**Charity Care**	**Medicaid Shortfalls**	**Other Govt Program Shortfalls**	**Subsidized Health Services**
**Post-policy slope change**	**See note**							
Post(t) × T(t)								
**Linear time trend (years**)	0.00806 ***	0.00797 ***	−0.000215	0.000307	−0.00136 *	0.00430 **	0.00149 **	0.00354 ***
T(t)	(0.00222)	(0.00231)	(0.000379)	(0.000517)	(0.000748)	(0.00169)	(0.000594)	(0.00122)
**Intervention**	−0.00378	−0.00112	−0.00220 **	−0.000469	−0.000557	−0.00178	−0.00101	0.00224
Post(t)	(0.00550)	(0.00554)	(0.00103)	(0.000608)	(0.00129)	(0.00384)	(0.00168)	(0.00298)
Median Income (USD)	−1.27 × 10^−6^ *	−1.10 × 10^−6^	7.82 × 10^−8^	−2.41 × 10^−7^	9.33 × 10^−7^ ***	−1.23 × 10^−6^ **	−8.46 × 10^−8^	−7.17 × 10^−7^ *
	(7.27 × 10^−7^)	(7.00 × 10^−7^)	(1.41 × 10^−7^)	(2.24 × 10^−7^)	(2.44 × 10^−7^)	(5.30 × 10^−7^)	(1.33 × 10^−7^)	(3.60 × 10^−7^)
Uninsured (%)	0.00356 ***	0.00382 ***	−0.000294	4.00 × 10^−5^	0.00136 ***	0.00106	0.000850 **	0.000548
	(0.00111)	(0.00113)	(0.000221)	(0.000184)	(0.000396)	(0.000900)	(0.000333)	(0.000722)
Herfindahl–Hirschman Index	−0.00362	−0.00366	0.00239	−0.00235	−0.00678	0.0124	0.00478	−0.0140
	(0.0272)	(0.0238)	(0.00220)	(0.00330)	(0.00467)	(0.0126)	(0.00488)	(0.0126)
Number of hospital beds	0.000131 **	0.000148 **	6.87 × 10^−6^	−2.40 × 10^−5^	−2.26 × 10^−5^	0.000151	7.12 × 10^−6^	1.23 × 10^−5^
	(5.76 × 10^−5^)	(6.33 × 10^−5^)	(2.01 × 10^−5^)	(2.12 × 10^−5^)	(4.26 × 10^−5^)	(9.39 × 10^−5^)	(1.13 × 10^−5^)	(3.21 × 10^−5^)
Constant	0.124 ***	0.0317	0.00592	0.0219 **	−0.0287 **	0.0460 **	−0.0140 *	0.0284
	(0.0458)	(0.0324)	(0.00752)	(0.00946)	(0.0125)	(0.0220)	(0.00784)	(0.0229)
Observations	672	672	672	672	672	672	672	672
R−squared	0.063	0.081	0.027	0.041	0.219	0.043	0.068	0.142
SOURCE Authors’ analyses of audited hospital financial data and community benefit spending from the Oregon Health Authority Hospital Reporting Program and Agency for Healthcare Research and Quality Social Determinants of Health Database. NOTES The sample includes all nonprofit hospitals in Oregon that have complete data from 2011 to 2022 (n = 56). Direct patient care community benefit (CB) spending includes direct patient care charity care, shortfalls from Medicaid and other government programs, and subsidized health services. Social Determinants of Health community benefit (CB) spending includes community health improvement and operations, cash and in-kind contributions, and community building activities. Research and Education CB spending includes research and health profession education. Results of regression analyses are presented, adjusted for hospital bed size and county-level characteristics including uninsurance rate, median household income, and Herfindahl–Hirschman Index. Intervention Post(t) is a dummy variable that equals 1 for year ≥ 2022, and 0 otherwise. The coefficient on Post(t) x T(t) (Post-policy slope change) cannot be estimated because data is available through 2022. All models control for hospital fixed effects. Standard errors are clustered on hospitals. *** *p* < 0.01, ** *p* < 0.05, * *p* < 0.1.								

## Appendix I

Interrupted Time-Series Estimates of the Association between Oregon’s Community Benefit Policies an Community Benefit Spending in 2021 Dollars.

	**Total Community Benefit**	**Direct Patient Care**	**SDOH**	**Research and Education**	**Charity Care**	**Medicaid Shortfalls**	**Other Govt Program Shortfalls**	**Subsidized Health Services**
**Post-policy slope change**	708,929	979,914	−428,943 **	157,959	−379,080	1.054 × 10^6^	331,641	−26,350
Post(t) × T(t)	(1.277 × 10^6^)	(1.189 × 10^6^)	(192,125)	(227,239)	(311,607)	(644,879)	(200,068)	(864,647)
**Linear time trend (years)**	279,174	127,744	−127,982	279,411	−81,433	−364,212	−91,077	664,467
T(t)	(948,922)	(942,362)	(160,513)	(242,095)	(307,300)	(584,048)	(175,129)	(703,478)
**Intervention**	−7.859 × 10^6^	−1.004 × 10^7^	4.175 × 10^6^ **	−1.997 × 10^6^	4.034 × 10^6^	−1.224 × 10^7^ **	−2.567 × 10^6^	732,222
Post(t)	(1.096 × 10^7^)	(1.016 × 10^7^)	(1.759 × 10^6^)	(2.275 × 10^6^)	(2.661 × 10^6^)	(6.020 × 10^6^)	(1.545 × 10^6^)	(7.197 × 10^6^)
Median Income (USD)	491.1 *	509.8 *	89.28 *	−107.9	114.3 **	442.3	29.34	−76.17
	(253.7)	(300.2)	(46.06)	(122.4)	(55.47)	(268.9)	(27.06)	(61.91)
Uninsured (%)	118,619	118,523	−57,735	57,831	241,710	−325,852	−22,274	224,939
	(511,247)	(486,947)	(61,091)	(63,782)	(169,113)	(305,403)	(76,847)	(424,164)
Herfindahl–Hirschman Index	−2.305 × 10^6^	−2.113 × 10^6^	104,521	−296,289	−748,575	−749,140	212,820	−828,234
	(5.470 × 10^6^)	(4.887 × 10^6^)	(529,437)	(628,270)	(644,497)	(2.659 × 10^6^)	(244,680)	(3.001 × 10^6^)
Number of hospital beds	330,869 ***	324,130 ***	6,985	−246.2	−3,300	252,355 **	20,519 **	54,556
	(71,976)	(75,289)	(7,678)	(5,012)	(26,415)	(106,945)	(8,039)	(34,649)
Constant	−3.697 × 10^7^ **	−4.366 × 10^7^ **	−2.441 × 10^6^	9.129 × 10^6^ *	−2.955 × 10^6^	−3.176 × 10^7^	−2.562 × 10^6^ *	−6.378 × 10^6^
	(1.652 × 10^7^)	(1.775 × 10^7^)	(2.136 × 10^6^)	(4.958 × 10^6^)	(4.805 × 10^6^)	(1.905 × 10^7^)	(1.375 × 10^6^)	(1.011 × 10^7^)
Observations	672	672	672	672	672	672	672	672
R−squared	0.383	0.355	0.114	0.022	0.057	0.283	0.177	0.169
SOURCE Authors’ analyses of audited hospital financial data and community benefit spending from the Oregon Health Authority Hospital Reporting Program and Agency for Healthcare Research and Quality Social Determinants of Health Database. NOTES Community benefit spending dollars were converted to 2021 dollars using the consumer price index from the United States Bureau of Labor Statistics. The sample includes all nonprofit hospitals in Oregon that have complete data from 2011 to 2022 (n = 56). Direct patient care community benefit (CB) spending includes direct patient care charity care, shortfalls from Medicaid and other government programs, and subsidized health services. Social Determinants of Health community benefit (CB) spending includes community health improvement and operations, cash and in-kind contributions, and community building activities. Research and Education CB spending includes research and health profession education. Results of regression analyses are presented, adjusted for hospital bed size and county-level characteristics including uninsurance rate, median household income, and Herfindahl–Hirschman Index. All models control for hospital effects. Standard errors are clustered on hospitals. *** *p* < 0.01, ** *p* < 0.05, * *p* < 0.1.								
